# Towards a better understanding and improved refinement of disordered crystal structures

**DOI:** 10.1107/S2052252521001573

**Published:** 2021-02-20

**Authors:** Peter Müller

**Affiliations:** a MIT Department of Chemistry, 77 Massachusetts Avenue, Building 2, Room 325, Cambridge, MA 02139-4307, USA

**Keywords:** quantum crystallography, crystal structures, disorder refinement, molecule-in-cluster optimizations

## Abstract

The new approach of Dittrich [*IUCrJ* (2021). **8**, 305–318] towards describing, understanding and modelling disorders is discussed.

Single-crystal X-ray diffraction, the gold-standard of structure elucidation, has become well established, even mainstream, and in every respect a mature method. Most challenges of routine structure determination can be considered overcome and even mostly automatized. Modern diffractometer software determines unit cells and designs data collection strategies with minimal user input. Similarly, the phase problem is solved and space groups are determined fairly automatically by software, and most routine structure refinements can be performed with a few clicks of the mouse in just minutes. Even twinning has lost its terror through the power of computing, perhaps with the exception of reticular-merohedral twins. The one remaining issue in routine crystal structure determination is the refinement of disorder. An appreciable amount of crystal structures show at least some disorder, if only in the solvent part, and crystallographers regularly spend most of their time tackling disorders, some of which can take days to parameterize properly.

A new approach towards describing, understanding and modelling disorders has been offered by Birger Dittrich in this issue of **IUCrJ** (Dittrich, 2021 ▸). Dittrich introduces the useful term ‘archetype structure’, which he defines as each distinguishable conformation of the molecule(s) in a crystal structure. A structure with just one simple two-component disorder gives rise to two archetype structures, one consisting of all ordered atoms plus the atoms of one disorder component, the other archetype consists of all ordered atoms plus the atoms of the other disorder component. A structure with more disorders gives rise to a larger number of archetype structures: two independent two-component disorders lead to four archetype structures, three independent two-component disorders to eight, and so forth. The crystal structure is then the superposition of all archetype structures.

Dittrich’s method takes each archetype structure and individually optimizes it computationally by means of a ‘molecule-in-cluster’ method (Dittrich, Chen *et al.* 2020 ▸), where the geometry of a molecule undergoes an energy minimization while taking into consideration a cluster of surrounding molecules as arranged in the crystal packing. From the optimized geometries, Dittrich derives restraints that he uses to assist a conventional least-squares refinement, the results of which can significantly improve the fit of the molecular model to the diffraction data. Constraints are automatically generated for the displacement parameters of proximate atoms to avoid over-parameterization.

The geometry restraints derived from the optimization step are, necessarily, direct restraints where set target values are specified for interatomic distances and angles. This is in contrast to relative restraints (also known as similarity restraints) where parameters within a structure are related to one another rather than to specific target values. Direct restraints are generally based on ‘outside information’, that is from methods unrelated to the actual diffraction experiment and the crystal structure at hand. Therefore, direct restraints are sometimes considered less elegant and relative restraints are typically preferred for the refinement of disorders (Müller, 2009 ▸). In the case of Dittrich’s method, however, the target values are not taken from some table found in a textbook, but are derived computationally, based on the actual crystal structure, which largely negates the arguments against direct and for relative restraints.

With larger structures and especially with structures suffering several independent disorders, Dittrich’s molecular models can become bulky. Describing an ordered atom as the overlay of two, four or even more identical positions with individual occupancies adding to 100% requires the use of constraints to allow convergence of the refinement. This is not a problem, however, since constraints, by their very nature, eliminate refined parameters and there is no practical difference between an atom described with three positional and nine thermal parameters, and the same atom split into several sites that are constrained to exhibit identical coordinates and anisotropic displacement parameters.

In addition to showing a way of improving the fit of disordered molecular models to the diffraction data, Dittrich’s paper also contributes to the general understanding of the disorder phenomenon. For example his four commonsense requirements for disorders are helpful: disorder can only occur if (*a*) the disorder components overlap well (*i.e.* they fit inside the same ‘shrink-wrap envelope’), (*b*) the charge distribution is similar for all components (*i.e.* the electrical potential on the surface of the envelope is roughly the same for all components), (*c*) inter- and intramolecular contacts (such as, for example, hydrogen bonds) made by all components are similar, and (*d*) the conformational energies of the individual disorder components are not too different from one another. In addition, Dittrich places disorder in context with molecular conformation, space group symmetry and diffuse scattering, which links many aspects of crystal structure determination in a way that has practical implementations, thus transcending mere intellectual exercise.

Finally, Dittrich’s method allows for an interesting way of distinguishing static from dynamic disorder. Theoretically, the former is introduced during crystallization and cannot be influenced by the temperature during the diffraction experiment while the latter corresponds to actual motion in the crystal, which can be ‘frozen out’ or at least reduced at lower temperatures. The above-mentioned energy requirement, combined with the energy differences derived from the individual molecule-in-cluster optimizations, can help in classifying a disorder as static or dynamic and even have implications for understanding and possibly predicting polymorphism, which is of vital importance in the world of pharmacology (Dittrich, Sever & Lübben, 2020 ▸).

It remains to be seen how and when this method will be implemented by programmers and accepted by the crystallographic community. In many cases, a carefully and expertly executed conventional modelling of disorders using mostly similarity restraints for geometry and anisotropic displacement parameters is not significantly inferior to the results of Dittrich’s method; however, the molecule-in-cluster approach offers a new and relatively easy to use tool that will likely be considered a standard method before too long.

## References

[bb1] Dittrich, B. (2021). *IUCrJ*, **8**, 305–318. 10.1107/S2052252521000531 PMC7924241 33708406

[bb2] Dittrich, B., Chan, S., Wiggin, S., Stevens, J. S. & Pidcock, E. (2020). *CrystEngComm*, **22**, 7420–7431.

[bb3] Dittrich, B., Sever, C. & Lübben, J. (2020). *CrystEngComm*, **22**, 7432–7446.

[bb4] Müller, P. (2009). *Crystallogr. Rev.* **15**, 57–83.

